# Investigation of Neuropathology after Nerve Release in Chronic Constriction Injury of Rat Sciatic Nerve

**DOI:** 10.3390/ijms22094746

**Published:** 2021-04-29

**Authors:** Szu-Han Chen, Chia-Ching Wu, Sheng-Che Lin, Wan-Ling Tseng, Tzu-Chieh Huang, Anjali Yadav, Fu-I Lu, Ya-Hsin Liu, Shau-Ping Lin, Yuan-Yu Hsueh

**Affiliations:** 1 Division of Plastic and Reconstructive Surgery, Department of Surgery, National Cheng Kung University Hospital, College of Medicine, National Cheng Kung University, Tainan 701, Taiwan; coldking9@gmail.com (S.-H.C.); bbar01@gmail.com (W.-L.T.); 2 Center of Cell Therapy, National Cheng Kung University Hospital, College of Medicine, National Cheng Kung University, Tainan 701, Taiwan; 3 International Research Center for Wound Repair and Regeneration, National Cheng Kung University, Tainan 701, Taiwan; joshccwu@mail.ncku.edu.tw; 4 Department of Cell Biology and Anatomy, College of Medicine, National Cheng Kung University, Tainan 701, Taiwan; cathyhuang1991@gmail.com (T.-C.H.); Anjali.yadav1607@gmail.com (A.Y.); 5 Division of Plastic and Reconstructive Surgery, Department of Surgery, Tainan Municipal An-Nan Hospital, China Medical University, Tainan 709, Taiwan; fredlinkgh@gmail.com; 6 Department of Biotechnology and Bioindustry Science, College of Bioscience and Biotechnology, National Cheng-Kung University, Tainan 701, Taiwan; fuilu@mail.ncku.edu.tw; 7 The iEGG and Animal Biotechnology Center, National Chung Hsing University, Taichung 402, Taiwan; 8 Department of Life Sciences, National Cheng Kung University, Tainan 701, Taiwan; yhliu@mail.ncku.edu.tw; 9 Institute of Biotechnology, National Taiwan University, Taipei 106, Taiwan; shaupinglin@ntu.edu.tw

**Keywords:** compressive neuropathy, chronic constriction injury, nerve release, decompression, neuroinflammation, neuropathic pain, rat sciatic nerve

## Abstract

Peripheral compressive neuropathy causes significant neuropathic pain, muscle weakness and prolong neuroinflammation. Surgical decompression remains the gold standard of treatment but the outcome is suboptimal with a high recurrence rate. From mechanical compression to chemical propagation of the local inflammatory signals, little is known about the distinct neuropathologic patterns and the genetic signatures after nerve decompression. In this study, controllable mechanical constriction forces over rat sciatic nerve induces irreversible sensorimotor dysfunction with sustained local neuroinflammation, even 4 weeks after nerve release. Significant gene upregulations are found in the dorsal root ganglia, regarding inflammatory, proapoptotic and neuropathic pain signals. Genetic profiling of neuroinflammation at the local injured nerve reveals persistent upregulation of multiple genes involving oxysterol metabolism, neuronal apoptosis, and proliferation after nerve release. Further validation of the independent roles of each signal pathway will contribute to molecular therapies for compressive neuropathy in the future.

## 1. Introduction

Compressive neuropathy is common in traumatic peripheral nerve disease [1]. Carpal tunnel syndrome, for example, is the most common compressive neuropathy of the upper extremities, with a prevalence of approximately 5% [2,3]. When the peripheral nerves undergo local compression, initial neurogenic symptoms of neuropathic pain develop, including paresthesia and dysesthesia, resulting in tingling, numbness, and burning sensations for patients of carpal tunnel syndrome. If the external compression forces persist, subsequent muscle weakness and atrophy will gradually develop with time, increasing in severity [4]. With the increase in local compression forces, ischemia of the surrounding connective tissue and nerves might trigger a subsequent cascade of nerve damage and prolonged neuroinflammation [5,6]. The initial histopathological changes of compressive neuropathy induce a breakdown of the blood–nerve barrier and subperineurial edema [7]. The persistent compression contributes to thickening of both epineurium and perineurium, and the myelinated fibers result in segmental demyelination accompanied by small unmyelinated fiber degeneration. Later on, the entire peripheral nervous system experiences extensive neuropathology, including proximal neuronal cell damage and distal Wallerian degeneration [8].

Surgical decompression remains the gold standard treatment for compressive neuropathy in clinical practice, through which a patient’s clinical symptoms can be relieved to some degree. However, the incidence of patients who develop persistent or recurrent symptoms after surgical decompression remains high, ranging from 1% to 32% [2,9]. Repeated surgical intervention will be needed in 5% to 10% of patients with recurrent or persistent symptoms [10,11]. The known causes affecting the failure rate of surgical decompression include incomplete release of the transverse carpal ligament, persistent tenosynovitis, prolonged compression, delayed diagnosis, and extensive external and internal neural scarring [12,13]. In this manner, the success rate of repeated surgery for recurrent and persistent carpal tunnel syndrome might be much lower than that of primary surgical decompression. In their systematic review and meta-analysis, Soltani et al. reported no improvement in symptoms for up to 47% of patients following repeated surgical decompression [10]. Moreover, patients still had residual neurogenic dysfunction, even though they felt satisfied with the improvement in symptoms after repeated decompression surgery [10,11].

The main initial pathology of compressive neuropathy is persistent mechanical compression, inducing prolonged and extensive neuropathology in the peripheral nerve system. Nerve damage then leads to the recruitment of macrophages for phagocytosis, which promotes neuroinflammation via expression of proinflammatory cytokines such as tumor necrosis factor (TNF)-α and interleukin (IL)-1β, followed by segmental demyelination and Wallerian degeneration [14,15,16]. However, details regarding the molecular mechanisms and expression of inflammatory signaling genes associated with recurrent or prolonged compressive neuropathy after surgical decompression remain unclear. In this study, we aimed to investigate the influence of time period after nerve release using our previously established controllable chronic constriction injury (CCI) model [17]. The associated neuropathology and sensorimotor impairment, as well as the underlying molecular signature of prolonged neuroinflammation were explored and systemically identified.

## 2. Results

### 2.1. Late Release of Chronic Nerve Constriction Results in Persistent Neuropathic Pain

**Figure 1:**
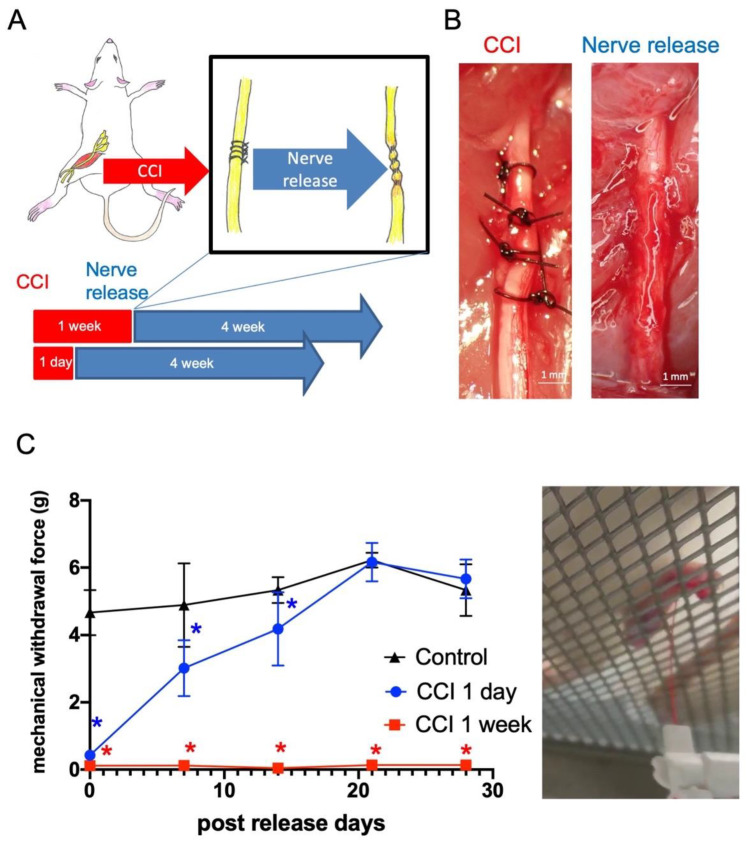
Chronic constriction injury model and sensory outcome. (**A**) Animal model of chronic constriction injury over ipsilateral sciatic nerve. Two time periods of constriction, followed by nerve release at 1 day and 1 week. (**B**) Controllable mechanical constriction forces were applied with four sutures. Nerve release was performed by neurolysis and removal of all sutures at 1 day or 1 week after constriction. Scale bar: 1 mm. (**C**) Mechanical allodynia was evaluated by Von Frey test over hind paw of rats. The mechanical withdrawal force (g) indicated that the forces that induced paw withdrawal by Von Frey test. (Data was presented with mean ± standard deviation, * indicated statistical significance as compared to control group; *p* < 0.05).

In order to investigate the neuropathy after nerve compression and decompression, we first validated the timing of constriction injury, followed by nerve decompression, to recapitulate the clinical neurolysis operation. Chronic constriction injury (CCI) was introduced by controllable forces (6 g string tension) upon the adult rat sciatic nerve, producing significant mechanical allodynia, moderate muscle weakness, and profound neuroinflammatory signals, as described in our previous work with non-release forces [17]. In this study, the release of nerve constriction forces, 1 day or 1 week after nerve injury, was performed by careful removal of the suture under microscopy (Figure 1A,B). Von Frey tests were performed every week, revealing that the withdrawal forces returned to normal 3 weeks after release in the CCI 1 day group. However, mechanical allodynia was persistently presented in the CCI 1 week group, demonstrating that irreversible damage was induced by 1 week of nerve constriction injury (Figure 1C).

### 2.2. Chronic Constriction Injury for 1 Week Produces Significant But Reversible Muscle Atrophy

**Figure 2:**
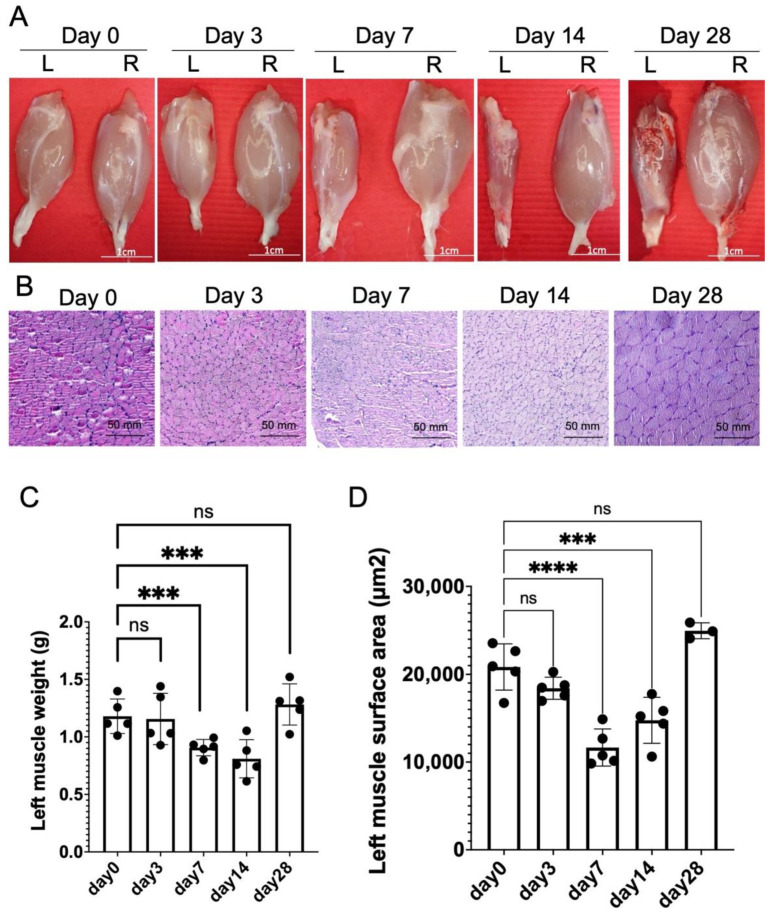
Gastrocnemius muscle evaluation by 1 week CCI. (**A**,**C**) Multiple time point analysis of gastrocnemius muscle at post nerve release day 0, 3, 7, 14 and 28 (L: left injured side; R: right uninjured side). Left gastrocnemius muscle weight demonstrated significant decrease till post release day 14, with recovery at day 28 after nerve release. (Scale bar: 1 cm. Data was presented with mean ± standard deviation, *** indicated *p* < 0.001, **** indicated *p* < 0.0001, ns indicated no significant difference) (**B**,**D**) Histology of affected side muscle fiber demonstrated decrease of left muscle fiber surface area from day 0 to day 14, with increase at day 28 after nerve release. (Scale bar: 50 mm. Data was presented with mean ± standard deviation).

Focusing on the model of CCI for 1 week (Figure 1), the influence of muscle mass after nerve release was also validated. After nerve release, the left gastrocnemius muscle weight post-release was reduced significantly from day 0 to day 14, with recovery at day 28 (Figure 2A,C). The surface area of left injured gastrocnemius muscle fiber also revealed significant atrophy from day 0 to 14, with recovery at day 28 after nerve release (Figure 2B,D).

### 2.3. Release of 1-Week Constricted Nerve Induces Delayed Axon Remyelination

**Figure 3:**
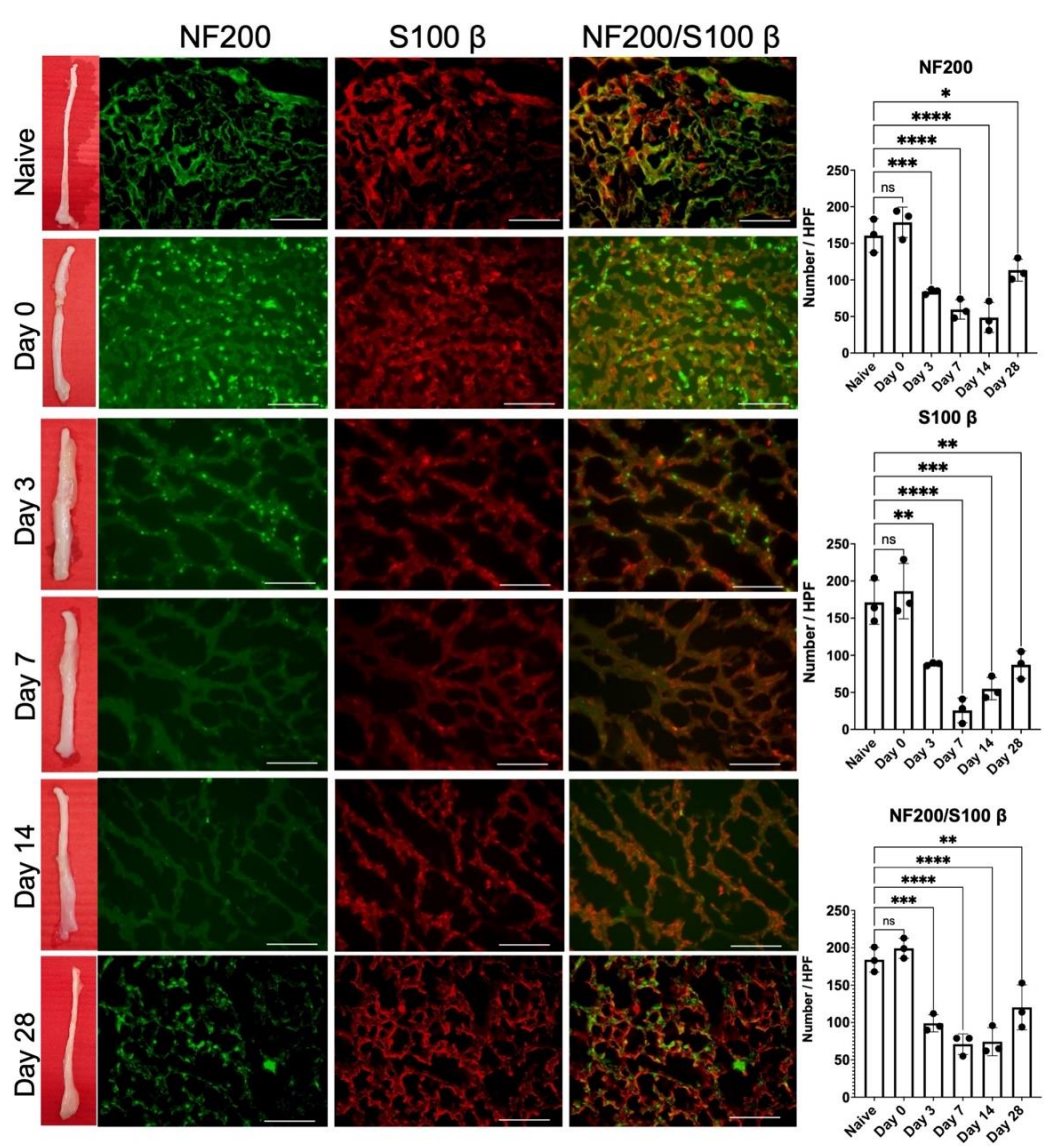
Immunohistochemical staining of injured sciatic nerve for axon myelination. Gross nerve image demonstrated apparent nerve constriction at nerve release day 0, followed by nerve swelling till day 14. No apparent nerve damage was observed at day 28. Mature axon staining with NF200 revealed significant decrease of signal intensity from day 0 to day 14, with partial increase at day 28. Similar pattern was also observed in Schwann cell staining with S100β along and double staining image. Double staining of NF200/S100β revealed degree of myelinated axon after nerve release. (n = 3 per timepoint; Naïve nerve: contralateral uninjured nerve; Scale bar: 50 μm; * indicated *p* < 0.05, ** indicated *p* < 0.01, *** indicated *p* < 0.001, **** indicated *p* < 0.0001, ns indicated no significant difference).

Since significant mechanical allodynia persisted even 28 days after nerve release (Figure 1), we further investigated axon regeneration following nerve release. From the gross picture of the injured sciatic nerve, we observed obvious nerve constriction at day 0, followed by perineural swelling from day 3 to 7 (Figure 3). From day 14, the appearance of injury to the nerve was revealed upon comparison with a naïve nerve that lacked significant nerve swelling, and constriction. Immunofluorescent staining of NF-200 and S100β in the injured nerve distal to the nerve injury site revealed a significant decrease in myelinated axons from day 0 to 14 post-release (Figure 3). Increase in remyelinated axons was apparent on day 28, which correlated with the recovery of muscle weight and muscle fiber surface area on day 28 (Figure 2C,D).

### 2.4. Release of 1-Week Constricted Nerve Demonstrated Prolonged Neuroinflammation of Injured Nerve and Dorsal Root Ganglion

**Figure 4:**
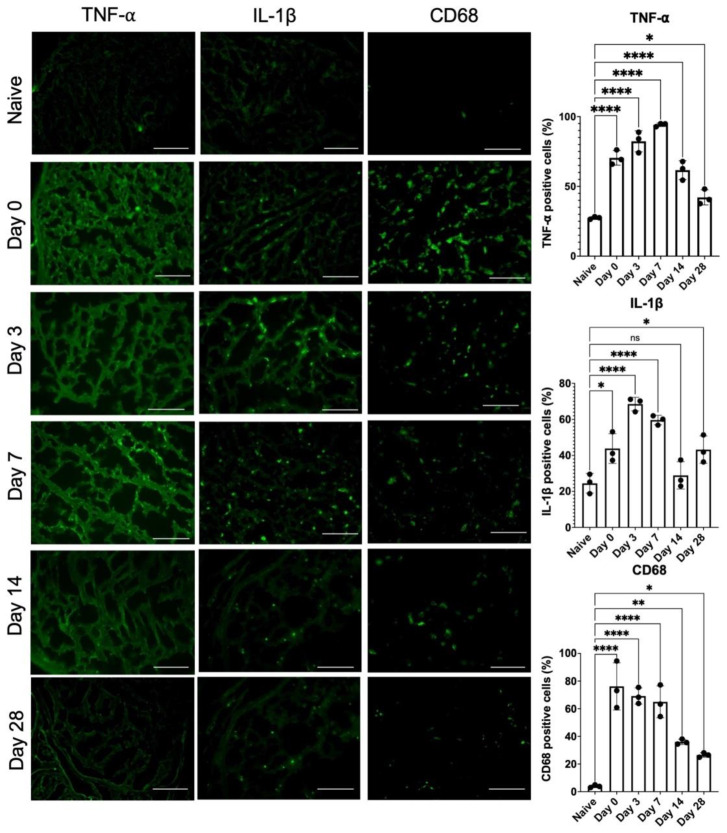
Immunohistochemical staining of injured sciatic nerve for neuroinflammation. Significant increase of signal intensity (TNF-α, IL-1β and CD68) was induced by nerve constriction injury at day 0, as compared to naïve nerve as baseline. After nerve release, the signal intensity decrease from day 0 to day 28. (n = 3 per timepoint; Naïve nerve: contralateral uninjured nerve; Scale bar: 50 μm; * indicated *p* < 0.05, ** indicated *p* < 0.01, **** indicated *p* < 0.0001, ns indicated no significant difference).

The neuroinflammation profiles and the underlying molecular signals were explored to identify factors correlated with persistent mechanical allodynia. From the distal nerve immunofluorescent staining, persistent inflammatory signals were observed in TNF-α and IL-1β from day 0 to 7 (Figure 4). After day 14, the inflammatory signals decreased but remained persistent on day 28 as compared with those in the naïve uninjured nerve. The immunofluorescent staining of CD68+ inflammatory cells also showed a similar trend in the injured nerve.

**Figure 5:**
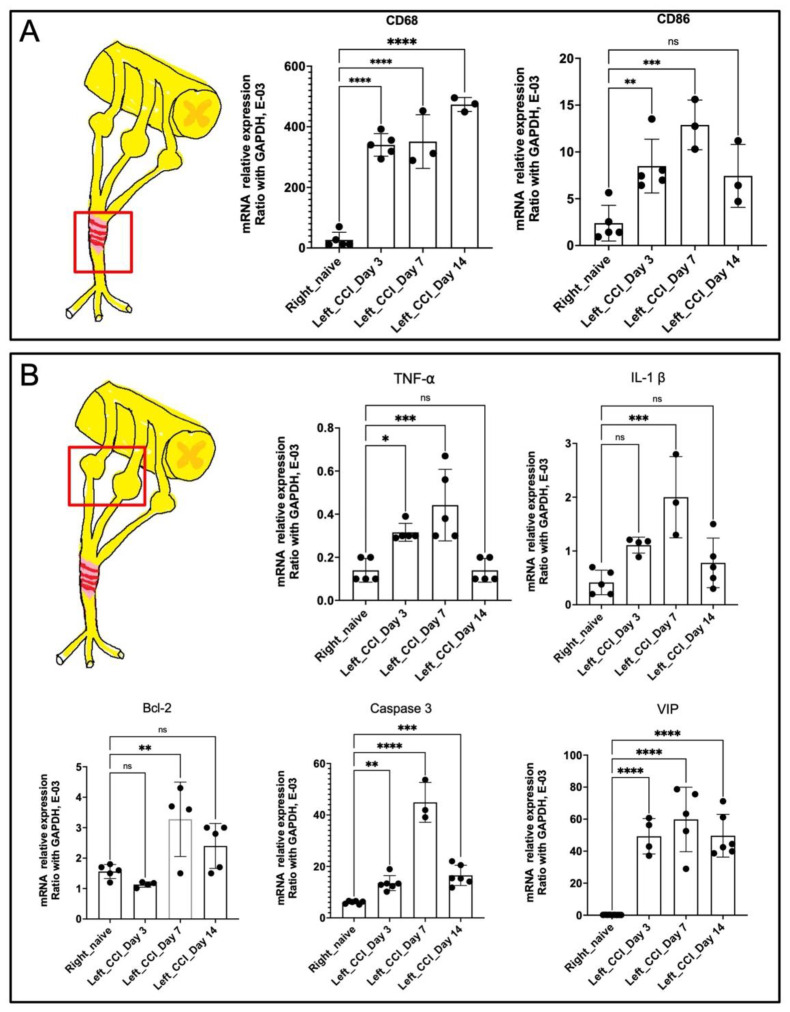
Quantitative PCR analysis of inflammatory gene among injured nerve and dorsal root ganglion. (**A**) Upregulation of CD68 and CD86 expression over injured nerve at post nerve release day 3, 7 and 14, as compared to uninjured nerve. (**B**) Upregulation of TNF-α, IL-1β, BCL-2, Caspase 3 and VIP expression over injured DRG at post nerve release day 3, 7 and 14, as compared to uninjured side. (Data was presented with mean ± standard deviation, * indicated *p* < 0.05; ** indicated *p* < 0.01, *** indicated *p* < 0.001, **** indicated *p* < 0.00001, ns indicated no significant difference).

Therefore, in order to explore the regulation of inflammatory signals at early time points (day 0–14), the expression of genes involved in inflammatory signaling was characterized in both the distal nerve and the dorsal root ganglion (DRG) using qPCR. In the injured nerve, the expression levels of CD68+ and CD86+ were both upregulated from day 3 to 14 (Figure 5A), indicating persistent inflammatory macrophage infiltration within the injured nerve. Regarding the inflammatory signals of ipsilateral DRG, upregulation of both TNF-α and IL-1β from day 3 to 7 was demonstrated, with downregulation on day 14 (Figure 5B). However, regarding cell apoptosis signaling, B-cell lymphoma (BCL)-2 and caspase-3 remained upregulated until day 14, indicating persistent neuronal damage. Moreover, prolonged upregulation of the pain-related signal, vasoactive intestinal polypeptide (VIP), was strongly correlated with persistent mechanical allodynia (Figure 1).

### 2.5. Nerve Release from CCI Revealed Bimodal Gene Regulation in Inflammatory and Innate Immune Profiling

**Figure 6:**
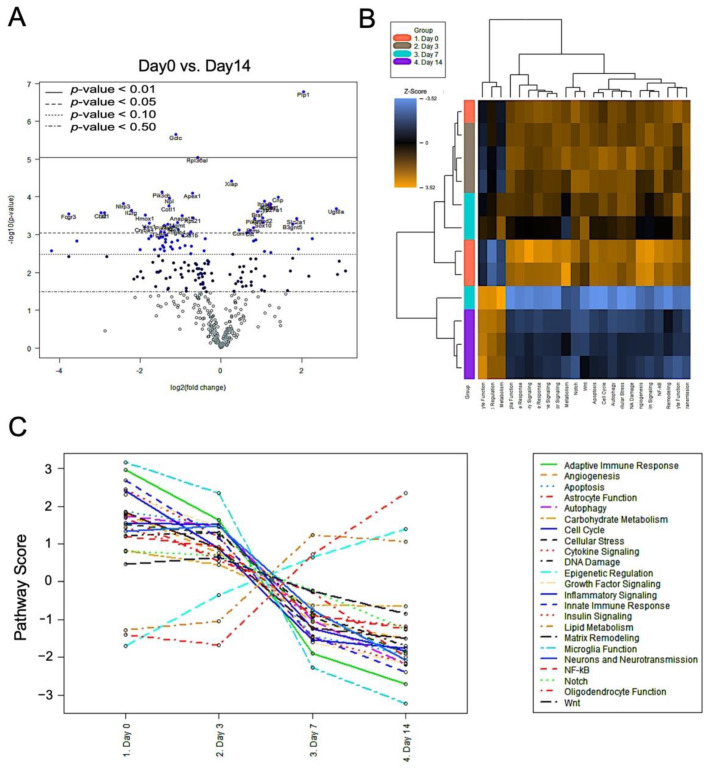
Genetic profiling of injured nerve by NanoString nCounter Neuroinflammation panel. (**A**) Volcano plot demonstrated distribution pattern of both upregulation and downregulation among established neuroinflammation panel, comparing post nerve release day 0 versus day 14. (**B**,**C**) Heat map distribution of 23 neuroinflammation pathways and processes among post nerve release day 0 to day 14. Significant upregulation of 20 inflammatory pathway in day 0–7, with downregulation at day 14. Reverse pattern presented among three pathways, including oligodendrocyte function, epigenetic regulation, and lipid metabolism. (n = 3 for each group).

To further investigate the molecular mechanism of prolonged neuroinflammation after nerve release in the CCI model, systemic gene expression profiling was performed to determine neuroimmune interactions. The injured nerves released from CCI were harvested, and the RNA of injured nerves from day 0 to 14 was isolated. The volcano plot demonstrated two distinct sets of gene profiles, i.e., upregulation and downregulation, when comparing expression between day 0 and 14 (Figure 6A). The genetic heatmap analysis among 23 neuroinflammation pathways demonstrated significant global upregulation in 20 regulatory pathways from neuroinflammatory panel on day 0, 3, and 7 post-release (Figure 6B). On day 14, the signals of these pathway were downregulation, and the results were in accordance with those for immunofluorescence staining of inflammatory factors TNF-α, IL-1β, and CD68 (Figure 4). However, three signal pathways involving oligodendrocyte function, epigenetic regulation, and lipid metabolism showed a reverse pattern of regulation, with upregulation seen after nerve release from day 0 to day 14 (Figure 6C).

**Figure 7:**
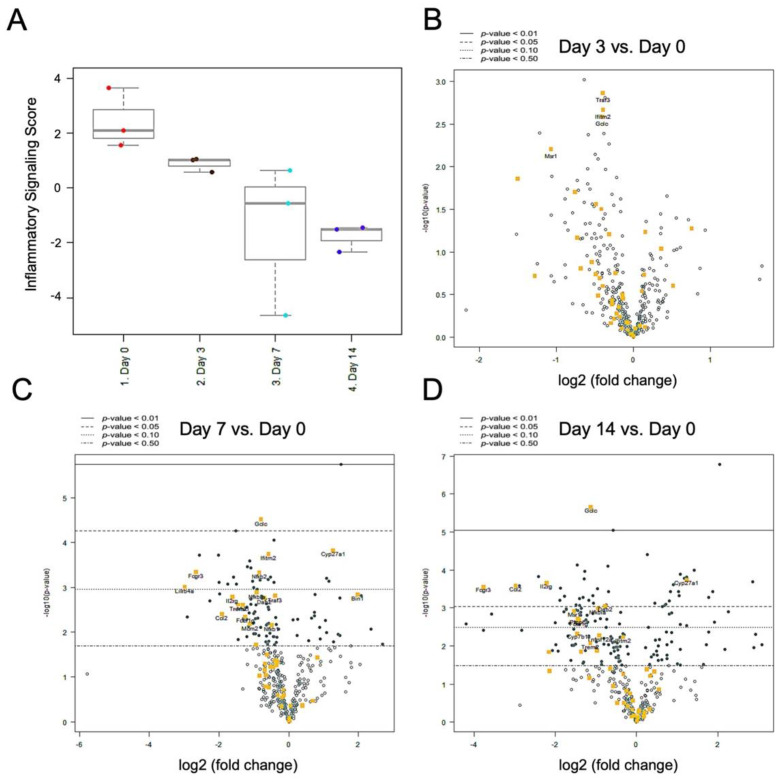
Differential gene expression in inflammatory signaling in injured nerve. (**A**) NanoString nCounter inflammatory signaling score revealed downregulation from day 0 to day 14. (**B**–**D**) Volcano plot of differential gene expression at day 3, day 7 and day 14, comparing with day 0. (n = 3 for each group).

The differential expression of inflammatory signaling showed overall downregulation from day 0 to 14 post-nerve release (Figure 7A). In comparison with their levels on day 0 as a baseline, Traf3, Ifitm2, Gclc, and Msr1 expression showed significant downregulation on day 3 (Figure 7B). On day 7, a large set of genes showed significant downregulation, including Gclc, Ifitm2, Fcgr3, Nfkb2, and Lilrb4a. However, two genes were upregulated on day 7, i.e., CYP27A1 and Bin1 (Figure 7C). On day 14, Gclc, Il2rg, Ccl2, and Fcgr3 remained significantly downregulated, while CYP27A1, conversely, remained upregulated (Figure 7D).

**Figure 8:**
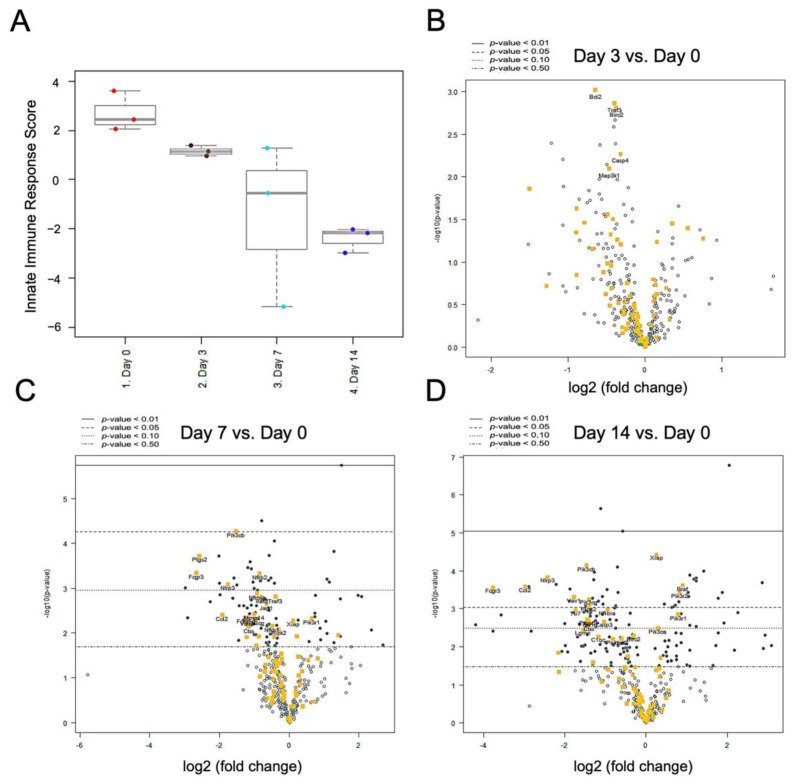
Differential gene expression in innate immune signaling in injured nerve. (**A**) NanoString nCounter innate immune signaling score revealed downregulation from day 0 to day 14. (**B**–**D**) Volcano plot of differential gene expression at day 3, day 7 and day 14, comparing with day 0. (n = 3 for each group).

The differential expression of innate immune signaling revealed a global downregulation pattern from day 0 to 14 (Figure 8A). Compared to day 0 as a baseline, Bcl2, Traf3, Birc2, Casp4, and Map3k1 first revealed significant downregulation on day 3 (Figure 8B). On day 7, a large set of signals showed significant downregulation, including Pik3cb, Ptgs2, Fcgr3, Nfkb2, and Nlrp3. However, two genes were upregulated on day 7, i.e., Pik3r1 and Xiap (Figure 8C). On day 14, Pik3cb, Nlrp3, Ccl2, Fcgr3, Vav1, Pik3cg, and Plcg2 remained significantly downregulated, while Xiap, Braf, and Pik3r1, by contrast, remained upregulated (Figure 8D).

## 3. Discussion

The CCI model is an animal model commonly and widely used to investigate nerve chronic compressive neuropathy [17,18]. Neuropathic pain and muscle weakness are persistent for 1 month after peripheral nerve injury [17]. These previous animal studies demonstrated that the persistent compression mechanical forces induced consistent neurologic dysfunction. The behavior impairments and functional loss in the CCI model are similar to the changes seen in compression neuropathy in humans. From our previous work, chronic controllable mechanical compression forces induced reliable and reproducible neurological deficits in rat sciatic nerve [17]. However, the applied compressive forces were permanent, without nerve release. In order to recapitulate the recurrent compressive neuropathy after primary nerve decompression, we investigated the neurological deficit following different durations of nerve constriction injury. Nerve release 1 day after controlled nerve constriction injury revealed significant mechanical allodynia on day 0, 7, and 14 post-release, but it returned to normal on day 21 (Figure 1C). This brief and reversible neurologic deficit was identical to the findings using the acute nerve crush or clip model, with spontaneous full recovery after injury [19,20]. Dableh et al. [5] reported that the tactile hypersensitivity induced by temporary mechanical compression can be spontaneously reversed by removing the implanted perineural cuff of the sciatic nerve. They revealed that removal of the cuff at 24 h after hypersensitivity induction led to full recovery to the baseline threshold, whereas removal of the cuff after 4 days demonstrated only partial alleviation. Pettersson et al. also found that early nerve decompression followed by conduit compression injuries was of great importance for sensorimotor recovery in rat when comparing the results for a 3-day vs. a 28-day compression period [21]. In this study, when the controllable mechanical compression was applied for 1 week, release of suture could not reverse the neurologic deficit, and the mechanical allodynia persisted (Figure 1C). The relative muscle weight and surface area also demonstrated progressive loss without significant recovery on day 28 post-nerve release (Figure 2). From the present evidence, 1 week nerve constriction injury is sufficient to induce irreversible mechanical allodynia and significant muscle atrophy, even after nerve release. The transition from mechanical-induced nerve injury to chemical-related neuropathology should be further investigated.

To further explore local inflammation within the injured nerve, the number of myelinated axons distal to the nerve constriction region was examined through immunofluorescence staining of NF200 and s100β. A significant decrease in signal corresponding to NF200/s100β double staining was observed on day 0, as compared with naïve uninjured nerve (Figure 3). Despite nerve release, the neuropathology of the injured nerve demonstrated a continuous process of demyelination until day 14. On day 28, the injured nerve returned to its original gross appearance without any signs of swelling nor perineurial fibrosis. Double staining of the nerve demonstrated partial remyelination of axons on day 28, which was compatible with the recovery of muscle mass (Figure 2). Similar findings were reported by Hu et al., demonstrating that axon remyelination was observed 6 weeks after nerve decompression in chronic sciatic nerve compression [22].

In addition, the inflammatory signals (TNF-α, IL-1β) and infiltration of CD68+ inflammatory cells also demonstrated a partial decrease after nerve release on day 3, whereas they remained persistent until day 28 (Figure 4). Ceyhan et al. demonstrated similar results, whereby chronic constriction release partially decreased TNF-α expression in the injured nerve [23]. Moreover, George et al. observed that thalidomide treatment alleviated CCI-induced neuropathic pain, which was associated with reduced sciatic endoneurial TNF-α content, but without any change in IL-β [24]. Orhan et al. also reported that the administration of sirolimus, an immunosuppressive antibiotic, significantly alleviated CCI-induced neuropathic pain associated with reduced TNF-α protein levels in the spinal cord. However, the spinal cord IL-1β and IL-6 protein levels were not affected [25]. The expression levels of TNF-α and IL-1β play independent roles in the development of CCI-induced neuropathic pain [26]. Several inflammatory cells, such as macrophages, participate in neuroinflammation and regeneration [27], which presented at the site of the lesion within 1 day and were recruited at their peak within 2 to 3 weeks [28]. Macrophages were responsible for phagocytosis within days of peripheral nerve injury, debris removal, growth factor production, and remodeling of the extracellular matrix of the distal nerve [29,30]. Pan-macrophage markers, such as CD68, indicate the presence of both phagocytic and releasing cytokines and chemokines via macrophage infiltration [31,32]. In the injured nerve, both CD68 and CD86 expression remained upregulated from day 3 to 14 (Figure 5A), which demonstrates the presence of specific inflammatory macrophage signals. Taken together, the sustained neuropathology (Figure 3) as well as the poor recovery of both sensory (Figure 1) indicate the presence of sustained neuroinflammatory signals (Figure 4 and Figure 5A) even after nerve release.

After peripheral nerve injury, the dramatic changes observed in the gene expression profiles within the dorsal root ganglia could be related to processes involving neuroinflammation signals, cell death, and neuropathic pain [33]. Inflammatory cytokines activated in the dorsal root ganglion after peripheral nerve injury might promote macrophage phagocytosis, Wallerian degeneration, and nerve regeneration [34]. The major proinflammatory response signals are TNF-α and IL-1β [33,34]. In this study, both TNF-α and IL-1β were upregulated in the dorsal root ganglion from day 3 to 14 post-nerve release (Figure 5B). In addition, nerve injury also induced higher expression of the proapoptotic factors Bcl-2 and caspase-3 in the dorsal root ganglion [35,36]. Previous investigators have demonstrated an increase in proapoptotic factors persisting for over 1 month after peripheral nerve injury [35,36]. Our study also showed the induction of Bcl-2 and caspase-3 gene expression in the dorsal root ganglion (Figure 5B). Moreover, an increase in the expression of neuropeptides (e.g., VIP), which contribute to the signals of neuropathic pain, could also be triggered in nerve constriction injury [33,37,38,39]. In this study, neuropathic pain was persistent after CCI, even 28 days after nerve release (Figure 1). The upregulation in VIP expression demonstrated a positive correlation with sustained induced mechanical allodynia (Figure 5B).

In order to explore the molecular pathways involved in sustained neuropathy after chronic constriction injury, differential mRNA expression was comprehensively surveyed using NanoString nCounter technology for gene profiling based on a panel of neuroinflammation genes. On day 14 after nerve release, mRNA within the injured nerve showed significant downregulation of a large set of inflammatory genes, whereas 32 genes remained upregulated (fold-change ≥ 2.0) (Figure 6). The genes that were downregulated from day 0 to 14 post-nerve release were involved in major neuroinflammatory pathways, such as adaptive immune response, cytokine signaling, apoptosis, autophagy, cellular stress, and DNA damage. The mRNA levels of three signal pathways involving oligodendrocyte function, epigenetic regulation, and lipid metabolism showed a reverse pattern from day 0 to 14, as compared to most of the neuroinflammatory signals.

The differential gene expression profile for specific pathways of inflammatory signaling and innate immune response showed that the overall mRNA expression level of both signaling pathways was downregulated from day 0 to 14 post-nerve release. However, several signaling genes showed the reverse trend, where upregulation in Cyp27a1, Xiap, Braf, and Pik3r1 was observed (Figure 7 and Figure 8). Cyp27a1 encodes one of the key catalyzing enzymes that oxidize steroid sidechains, converting cholesterol into dihydroxycholesterols such as 7a,25-dihydroxysterol (7a,25-OHC) and 7a,27-OHC [40]. Such oxidized derivatives of cholesterol, named oxysterols, have been demonstrated to play a role in several forms of neuroinflammation disease [41,42,43]. In the peripheral nerve system, polyneuropathy is a clinical manifestation in cerebrotendinous xanthomatosis, which is a rare autosomal recessive disorder of bile-acid metabolism caused by deficiency of the sterol 27-hydroxylase (Cyp27a1) gene [44,45]. The X-linked inhibitor of apoptosis protein (Xiap) encoded by the Xiap gene is a member of the inhibitor of apoptosis family of proteins. In addition to blocking apoptosis, this protein modulates many other signaling processes, including the nuclear factor kappa-light-chain-enhancer of activated B cells (NF-κB) pathway and inflammatory responses. In rat peripheral nerve injury, the upregulated expression of Xiap may decrease neuronal cell apoptosis, resulting in subsequent peripheral nerve regeneration [46,47]. Braf (alternately referred to as v-raf murine sarcoma viral oncogene homolog B1) is a proto-oncogene involved in the mitogen-activated protein kinase (MAPK) pathway. In the peripheral nerve system, Braf genetic alterations are found involved in the formation of sporadic schwannomas and malignant peripheral nerve sheath tumors [48,49]. Pik3r2 is a protein-coding gene that encodes the regulatory component of phosphoinositide 3-kinase (PI3K), which has a diverse range of cell functions including proliferation and survival. In peripheral nerve injury, the related PI3K/protein kinase B (Akt) pathway might be involved in the Schwann cell response to excessive oxidative stress-induced apoptosis [50]. Further validation regarding the pivotal role of the abovementioned regulating genes responsible for prolonged neuroinflammation is needed in the future.

## 4. Materials and Methods

### 4.1. Animals and Surgical Procedures

Animal protocols and surgical procedures such as animal housing and care were approved by the Laboratory Animal Center and Institutional Animal Care and Use Committee (IACUC; Protocol No. 108032) at National Cheng Kung University (Tainan, Taiwan). Adult Sprague–Dawley male rats with 8-week age, weighing 170–220 g, were used in this study. All surgery procedures were sterile; the surgical work surface was sterilized with 70% ethanol (EtOH), and sterile instruments and gauze were used. The average time to perform an operation was approximately 15 min. The animals were anesthetized with 2.5% isoflurane. All surgeries were performed under general anesthesia induced by an intraperitoneal injection of zoletil 50 (1 mL/kg) and 2% xylazine (0.1 mL/kg; Rompun; Bayer, Leverkusen, Germany). All rats were euthanized using standard IACUC-approved procedures.

All rats received two surgical procedures. First, the CCI model was created as described in our previous studies on the left sciatic nerve [17]. The left sciatic nerve was dissected from the surrounding tissues and exposed at the mid-thigh level. The area proximal to the trifurcation of the sciatic nerve was freed from the adhering tissue. Four ligatures with 5-0 nylon (Ethicon US, Bridgewater, NJ, USA) were tied around the sciatic nerve, with approximately 1 mm between each ligature. The left sciatic nerve was compressed 7 days after the first surgical procedure, and these CCI rats received a secondary operation. The injured left sciatic nerve was exposed again, and the adhesion connective tissue around the injured nerve and four nylon ligatures were carefully removed. After functional assessment for another month, the bilateral gastrocnemius muscles, sciatic nerves, and dorsal root ganglions were harvested for further investigation.

### 4.2. Sensory Assessment

The mechanical hyperalgesia was measured using the von Frey test, which dictates that the threshold force for paw withdrawal decreases dramatically after nerve injury. Spontaneous foot lifting may provide a behavioral measure of spontaneous pain. The rats stood on an elevated platform made of a wide-gauge wire mesh. From below, a von Frey hair was inserted up through the holes in the mesh and poked through the undersurface of a hind paw. At threshold, the animal would respond by flicking its paw away from the hair. These CCI surgical decompression rats underwent von Frey tests every week for 1 month. We analyzed the mechanical hyperalgesia in the CCI surgical decompression group compared with the control rats that received neurolysis alone.

### 4.3. Wet Muscle Evaluation and Histological Analysis

In rats, the gastrocnemius muscle is the largest muscle innervated by the sciatic nerve and begins to atrophy after nerve injury. To assess nerve reinnervation, the left gastrocnemius muscle weight and fiber surface area were immediately measured after the rats were sacrificed. The gastrocnemius muscles (excluding the soleus muscles) of both limbs were harvested from their bony attachments. Immediately after the measurement of muscle weight, the muscle tissues were fixed, and a histological assessment of the nerve tissues was then performed. Hematoxylin and eosin (H&E) staining was used to examine the morphology of muscle fibers.

### 4.4. Hematoxylin and Eosin Staining

Several histological stains were performed to observe the tissue morphology and protein expression. The paraformaldehyde-fixed muscle segments were dehydrated and then embedded in paraffin. The muscle tissues were sectioned transversely at 4–5 μm. Hematoxylin and eosin (H&E) staining was then applied to observe tissue morphology. To manually measure the muscle surface area (mm^2^), the margin of individual muscle fiber were marked using the ImageJ software.

### 4.5. Immunofluorescent Staining (IF)

Immunofluorescent staining was performed to detect specific expression patterns exhibited by proteins. The primary antibodies used were anti-neural filament heavy chain (NF200, N0142, 1:200; Sigma-Aldrich, St. Louis, MO, USA); s100 beta (ab52642, 1:200; Abcam, Cambridge, MA, USA), CD68 (ab125212, 1:200; Abcam, Cambridge, MA, USA), TNF-alpha (ab205587, 1:200; Abcam, Cambridge, MA, USA), and IL-1beta (GTX74034, 1:200; Genetex, Irvine, CA, USA). The tissue was embedded in OCT (Tissue-Tek^®^, Sakura Finetek Inc, Torrence, CA, USA) and deep-frozen until use. Nerves were cryo-sectioned at 10 μm and mounted in Mowiol^®^ (Sigma-Aldrich, St. Louis, MO, USA). Slices were visualized and photographed under fluorescence microscopy (BX61, Olympus, Tokyo, Japan).

### 4.6. RNA Preparation and Complementary DNA (cDNA) Synthesis

Total RNA was extracted from the nerves and DRG tissues of rat with TRIzol (Sigma-Aldrich, St. Louis, MO, USA), according to the manufacturer’s instructions. Total RNA was precipitated with 2-propanol, washed twice with 75% ethanol, and resuspended in a suitable volume of diethylpyrocarbonate (DEPC)-treated water. The total RNA concentration was determined by measuring the optical density (OD) values of the samples at 260 nm. To prepare first-strand cDNA, mRNA was reserve-transcribed with the reverse transcriptase enzyme (ImProm-II™ Reverse Transcriptase, Promega, Madison, WI, USA) in a reaction mixture (20 mL in total).

### 4.7. Quantitative PCR

Primers were designed for CD68, CD86, TNF-α, IL-1β, BCl-2, caspase-3, VIP, and glyceraldehyde 3-phosphate dehydrogenase (GAPDH). GAPDH was used as a reference gene. Primer sequences are given as follows:
**Name****Forward Primer/Reverse Primer**CD685′-CTGTTGCGGAAATACAAGCA-3′ 5′-GGCAGCAAGAGAGATTGGTC-3′CD865′-CCTCCAGCAGTGGGAAAC-3′ 5′-GTAGGTTTCGGGTATCCTTGC-3′TNF-α5′-TACTCCTCAGAGCCCCCAAT-3′ 5′-TCGTGTGTTTCTGAGCATCG-3′IL-1β5′-CTGTGACTCGTGGGATGATG-3′ 5′-TCCATTGAGGTGGAGAGCTT-3′BCl-25′-ACTCTTCAGGGATGGGGTGA-3′ 5′-TGACATCTCCCTGTTGACG-3′Caspase 35′-CCGACTTCCTGTATGCTTACTC-3′ 5′-CAGGGAGAAGGACTCAAATTC-3′VIP5′-CAGAAGCAAGCCTCAGTTCC-3′ 5′-GCCTGTCATCCAACCTCACT-3′GAPDH5′-TGGCCTCCAAGGAGTAAGAA-3′ 5′-TGTGAGGGAGATGCTCAGTG-3′

To analyze the mRNA levels, quantitative PCR was performed using the SYBR PCR Master Mix (GoTaq^®^ Green Master Mix, ProMega, Madison, WI, USA) and StepOnePlus™ System (ThermoFisher Scientific, Waltham, MA, USA). The quantitative PCR conditions were as follows: 10 min at 95 °C, followed by 40 cycles of 15 s at 95 °C and 30 s at 60 °C. The 2^−^^∆∆CT^ method was used to analyze quantitative PCR data.

### 4.8. NanoString nCounter Technology for Gene Profiling

Extracted RNA from the injured nerve was hybridized with a neuroinflammation panel of 770 barcoded probes (NanoString Technologies, Seattle, WA, USA) specific for genes associated with macrophage phenotype and a function involving peripheral nerve repair. Three biological replicates per condition were evaluated. Standard NanoString protocols were followed as per the manufacturer’s instructions. RNA sample quality was confirmed by spectrophotometry (QuickDrop SpectraMax, Molecular Devices, San Jose, CA, USA) to determine concentration and chemical purity (A260/230 and A260/280 ratios) and with a Bioanalyzer (model 2100, Agilent Technologies, Santa Clara, CA, USA) to determine RNA integrity; degraded samples were replaced with an alternative sample if available [51,52].

From the NanoString nCounter analysis, pathway scores condense each sample’s gene expression profile into a small set of pathway scores. An experiment can then be explored through the lens of pathway scores instead of in the much higher-dimension lens of gene expression values. Pathway scores are fit using the first principal component of each gene set’s data. They are oriented such that increasing score corresponds to mostly increasing expression (specifically, each pathway score has positive weights for at least half its genes). Summary plots explore the joint behavior of pathways, and Covariates plots compare pathway scores to covariates. Pathway analysis was also conducted to determine overall changes in pathways based on the first principal component of the targets within a pathway as annotated by NanoString (NanoString User Manual 10030-03). Direction of pathway change (up- or downregulated) was determined by cross referencing the pathway score with the corresponding volcano plot for that pathway. Summary pathway score plot colors are based on calculated scores and are represented as downregulation (blue) to upregulation (orange).

### 4.9. Statistical Analysis

General statistical analysis was used for experiment design and data analysis. The sample size necessary to detect a significant effect was estimated using Power and Precision statistical software (Biostat, Englewood, NJ, USA) with the following information: minimum significant effect to be detected, data variation, power (0.8), and type I error rate (0.05). For two-sample comparisons, Student’s *t*-test was used. For multiple-sample comparison, analysis of variance (ANOVA) was performed to detect whether a significant difference existed between groups with different treatments, and a post hoc Dunnett’s multiple comparison was used for post-hoc analysis to identify where the differences existed. *p* ≤ 0.05 was used to indicate significant differences between samples (two-tailed).

## 5. Conclusions

Peripheral neuropathology resulting from chronic constriction injury has a great impact, with irreversible mechanical allodynia and severe muscle mass atrophy 1 week after nerve constriction. Nerve release only results in partial recovery of the axon remyelination and neuroinflammation of the nerve and DRG. The genetic profiling of local nerve inflammation allowed exploring the underlying mechanisms, in which we identified potential molecular targets involved in oxysterol metabolism, neuronal apoptosis, and proliferation. Further genetic modification research should be conducted to validate the pivotal roles of these targets in sustained neuroinflammation and neuropathology.

## Data Availability

The data presented in this study are available in this manuscript.
